# RETRACTION: A Systematic Review of Nurses' Practice and Related Factors Toward Pressure Ulcer Prevention

**DOI:** 10.1111/iwj.70597

**Published:** 2025-04-21

## Abstract

**RETRACTION:** VajargahP. G. , MollaeiA. , FalakdamiA. , TakasiP. , MoosazadehZ. , EsmaeiliS. , ZeydiA. E. , and KarkhahS. , “A Systematic Review of Nurses' Practice and Related Factors Toward Pressure Ulcer Prevention,” International Wound Journal 20, no. 6 (2023): 2386–2401, 10.1111/iwj.14062.36543328 PMC10333028

The above article, published online on 21 December 2022, in Wiley Online Library (http://onlinelibrary.wiley.com/), has been retracted by agreement between the journal Editor in Chief, Professor Keith Harding; and John Wiley & Sons Ltd. Following an investigation by the publisher, all parties have concluded that this article was accepted solely on the basis of a compromised peer review process. In addition, further investigation by the publisher found that the methods section provided insufficient information for the study selection and analysis. The investigation also found that the results and discussion are not properly supported by the data presented. The editors have therefore decided to retract the article. The authors disagree with the retraction.

**RETRACTION:** P. G. Vajargah , A. Mollaei , A. Falakdami , P. Takasi , Z. Moosazadeh , S. Esmaeili , A. E. Zeydi , and S. Karkhah , “A Systematic Review of Nurses' Practice and Related Factors Toward Pressure Ulcer Prevention,” International Wound Journal 20, no. 6 (2023): 2386–2401, 10.1111/iwj.14062.36543328 PMC10333028

The above article, published online on 21 December 2022, in Wiley Online Library (http://onlinelibrary.wiley.com/), has been retracted by agreement between the journal Editor in Chief, Professor Keith Harding; and John Wiley & Sons Ltd. Following an investigation by the publisher, all parties have concluded that this article was accepted solely on the basis of a compromised peer review process. In addition, further investigation by the publisher found that the methods section provided insufficient information for the study selection and analysis. The investigation also found that the results and discussion are not properly supported by the data presented. The editors have therefore decided to retract the article. The authors disagree with the retraction.

